# Contributions of childhood peer victimization and/or maltreatment to young adult anxiety, depression, and suicidality: a cross-sectional study

**DOI:** 10.1186/s12888-021-03354-4

**Published:** 2021-07-14

**Authors:** Melissa Macalli, Massimiliano Orri, Christophe Tzourio, Sylvana M. Côté

**Affiliations:** 1 University of Bordeaux, Inserm, Bordeaux Population Health Research Center, UMR 1219, F-33000 Bordeaux, France; 2 McGill Group for Suicide Studies, Douglas Mental Health University Institute and Department of Psychiatry, McGill University, Montreal, QC Canada; 3 CHU de Bordeaux, F-33000 Bordeaux, France; 4 School of Public Health, University of Montreal, Montreal, QC H3T 1J4 Canada

**Keywords:** Peer victimization, Childhood maltreatment, Mental health, Anxiety, Depression, Suicidality, Young adults

## Abstract

**Background:**

Childhood maltreatment and peer victimization are major risk factors for depression and suicidal behavior. Furthermore, childhood maltreatment increases the risk of peer victimization. Our objective was to distinguish between the contributions of parental maltreatment and peer victimization to the development of mental health problems in young adulthood. Specifically, we tested whether peer victimization alone or in combination with parental maltreatment before 18 years old was associated with anxiety, depression, and suicidal thoughts and behaviors at age 21 years.

**Methods:**

We analyzed data collected from questionnaires administered in the i-Share (Internet-based Students’ Health ResearchEnterprise) study in France from February 2013 to September 2019 (*N* = 2271 participants). We performed multinomial and binary logistic regression analyses to assess the single and combined contributions of childhood peer victimization and parental maltreatment to anxiety, depression, and suicidality in adulthood.

**Results:**

Nearly one third of students (28.8%) reported at least one mental health problem; 29.8% reported peer victimization alone; 7.5% reported parental maltreatment alone; and 10.3% reported both parental maltreatment and victimization. In multivariate models, compared to participants that did not experience maltreatment or peer victimization, those that experienced peer victimization alone were more likely to report anxiety (adjusted odds ratio [aOR]: 1.90; 95% CI: 1.50–2.40), depression (aOR: 1.95; 95% CI: 1.46–2.60), or suicidal ideation, without (aOR: 1.62; 95% CI: 1.26–2.09) or with a suicide attempt (aOR: 2.70; 95% CI: 1.51–4.85). Similar associations were observed for participants that experienced maltreatment alone. Participants that experienced both maltreatment and peer victimization were at increased risk of depression (aOR: 2.63; 95% CI: 1.79–3.86) and suicidal ideation, with (aOR: 9.19; 95% CI: 4.98–16.92) and without a suicide attempt (aOR: 2.64; 95% CI: 1.86–3.76).

**Conclusions:**

Separate and combined exposures to parental maltreatment and peer victimization in childhood or adolescence were associated with increased risks of anxiety, depression, and suicidal behaviors. Peer victimization appeared to play a specific role in mental health disorders that were not otherwise explained by polyvictimization. Currently, peer victimization is a frequent, but avoidable type of child abuse; therefore, these findings have implications for policies for preventing and dealing with peer victimization.

## Background

Maltreatment and peer victimization in childhood are recognized as important risk factors for mental health problems worldwide [1, 2], including anxiety, depression, and suicidal behavior [3–8]. Child maltreatment includes all types of physical and/or emotional ill-treatment, sexual abuse, neglect, or negligence, which may result in actual or potential harm to the child’s health, survival, development, or dignity. Child maltreatment is generally perpetrated in the context of a relationship where the perpetrator holds responsibility, trust, or power over the child, such as the parent-child relationship [1]. The estimated prevalence of child maltreatment varies, depending on the country, the definition used, and the assessment methods. A previous review of a series of meta-analyses showed that the overall estimated prevalence rates of self-reported child maltreatment were 22.6%, for physical abuse, and 36.3%, for emotional abuse [9]. In high-income countries, such as the UK and the USA, the prevalence varied from 9 to 12% [10–12]. Unfortunately, reliable statistics are missing for a large number of countries – including France [13]. Maltreatment by peers (i.e., victimization) during childhood is a multifaceted experience, defined as harm caused by peers acting outside the norms of appropriate conduct [14]. The estimated prevalence of peer victimization varies, depending on the samples, the age of the cohort, and the methodology [15]. According to the World Health Organization, across 38 countries or regions, one in three children reported being bullied (a form of peer victimization involving an imbalance of power between the victim and the perpetrator), but the prevalence declined after the age of 11 years old [2].

Strong evidence from previous studies has suggested that children that experience maltreatment are at a higher risk of being victimized by their peers [14, 16–20]. Moreover, experiencing both forms of interpersonal violence is associated with an increased risk of mental disorders [21], compared to experiencing either maltreatment or peer victimization alone. Given the link between maltreatment and peer victimization and their long-term effects on mental health outcomes, it is relevant to investigate whether peer victimization might be associated with mental health problems, independent from maltreatment. Conversely, failing to take into account maltreatment when investigating associations between victimization and mental health problems could also obscure the potential combined effect of victimization and maltreatment on mental health outcomes [22]. Few previous studies have analyzed the separate and combined effects of peer victimization and maltreatment on mental health problems.

In the present study, our objective was to distinguish between these two types of interpersonal violence during childhood and their effects on mental health problems in young adulthood. Specifically, we tested whether peer victimization alone or in combination with parental maltreatment, experienced before 18 years old, was associated with anxiety, depression, and suicidal thoughts and behaviors (STB) at age 21 years, in a young adult French population.

## Methods

### Design, study population, and data collection

Our study sample comprised participants of the ongoing, Internet-based Students’ Health Research Enterprise (i-Share) project, a prospective, population-based study of volunteer students in French-speaking universities and higher education institutions. Enrollment in the i-Share project started in 2013; to be eligible, a student had to be officially registered at a University or higher education institute, at least 18 years of age, able to read and understand French, and provide informed consent for participation. The i-Share enrollment procedure was described previously [23].

The self-administered baseline questionnaire collected sociodemographic characteristics, health information, personal and familial histories, living conditions, and consumptions. For this cross-sectional retrospective study, participants completed the baseline questionnaire and a complementary questionnaire, which was proposed after inclusion to the study. Student volunteers received compensation of a 20-euro gift card for completing the supplementary questionnaire, part of which included questions about childhood adversity. For the present study, we retrieved data from a sample of students that were included in the i-Share cohort study between February 2013 and September 2019, had participated in the complementary questionnaire, and had data available on the outcomes of interest.

### Measures

#### Outcomes

- *Anxiety*. Participants completed the Spielberger State-Trait Anxiety Inventory (STAI-Y) [24]. Scores ranged from 20 to 80 points, and higher scores indicated greater anxiety. Cut offs that ranged from 39 to 55 have been suggested for detecting clinically relevant symptoms of anxiety [25, 26]. In our sample of young adults, we set the threshold to the third quartile. Thus, we defined “anxiety” as a binary variable with a STAI-Y score of 54 or higher.
- *Depression.* Participants completed the 9-item Patient Health Questionnaire (PHQ-9), which is a reliable, valid measure of depression severity over the preceding 2 weeks. Depression severities were graded according to PHQ-9 scores: 5 indicated mild depression, 10 indicated moderate depression, 15 indicated moderately severe depression, and 20 indicated severe depression. The ‘depression’ outcome was a binary variable, defined as a PHQ-9 score of 15 or higher, which reflected moderate to severe symptoms [27].
- *Suicidal thoughts and behaviors*. The baseline questionnaire included an STB assessment with single-item questions about suicidal thoughts during the last 12 months and lifetime suicide attempts. Suicidal thoughts were investigated with the question: “In the last 12 months, how often have you thought of attempting suicide (had suicidal ideation)?” Participants selected one of three possible responses: (1) no suicidal thoughts, (2) occasional suicidal thoughts, and (3) frequent suicidal thoughts. Suicide attempts are investigated with the question: “During your lifetime, have you attempted suicide?”. The variable “suicidal thoughts and behaviors” was divided into three modalities: “no” for no STBs (reference group); “suicidal thoughts without a suicide attempt” for occasional or frequent suicidal thoughts in the last 12 months, but no lifetime suicidal attempt; and “suicidal thoughts and suicide attempt” for occasional or frequent suicidal thoughts in the last 12 months and at least one lifetime suicide attempt.
- *Any mental health problems*. Participants with positive scores for depression, anxiety, or STBs were considered to have ‘any mental health problem’ (‘yes’), and these were compared to participants who reported none of these problems (‘no’).

#### Exposure

We assessed maltreatment and/or peer victimization using 17 questions from the Childhood Trauma Questionnaire [28]. Physical and psychological maltreatments were investigated with the following questions, respectively: 1) In your childhood or adolescence, did you feel that you were physically abused (beatings, physical punishment...) by your parents? 2) In your childhood or adolescence, did you ever feel like you had been psychologically or emotionally abused (unfair and frequent criticism, mockery, insults, humiliation, etc.) by your parents? Peer victimization was investigated with the question: In your childhood or adolescence, were you harassed by other children (i.e., regularly insulted or mocked or hit?) For all these questions, the answers “Never” and “Rarely” were coded as “No”, and the answers “Sometimes”, “Often”, and “Very often” were coded as “Yes”.

From these data, we defined an exposure variable for maltreatment and/or peer victimization. This variable had four categories: “None” for participants that answered no to all questions; “Peer victimization only” for participants that only answered yes to peer victimization; “Maltreatment only” for participants that only answered yes to maltreatment; and, “Both” for participants that answered yes to both maltreatment and victimization.

#### Covariates

We searched the literature to identify potential confounders related to childhood adversities that were associated with both mental health outcomes and the exposure variable. Thus, the following self-reported covariates were considered in the analyses: age, sex (male, female), parental divorce or separation (yes, no), parental depression or anxiety history (yes, no), parental alcohol abuse history (yes, no), parental education level (university studies, non-university studies), and a difficult economic status in childhood (yes, no).

### Statistical analyses

We first described the overall study sample, and grouped participants according to the categories of the exposure variable (i.e., none, peer victimization only, maltreatment only, or both maltreatment and peer victimization). Continuous variable “age” is expressed as the mean ± standard error. Categorical variables are described as the count and proportion (%). The Kruskal-Wallis test was used to compare distributions of age in the exposure variable groups. Proportions were compared with the Chi-square test.

We evaluated maltreatment and/or peer victimization associations with other binary outcomes (i.e., “anxiety”, “depression”, or “any mental health problems”) by performing binary logistic regression analyses. The results are expressed as adjusted odds ratios (aORs) with 95% confidence intervals (CIs). Because STB was a categorical variable with three modalities, we constructed multinomial logistic regression models to assess relationships between STBs and exposures. Model convergences were checked. The assumption of linearity of the logit was tested for the continuous variable, age, in each model. The fully adjusted analyses took into account all selected covariates. In each model, to account for missing information on covariates, we used the multiple imputation by chained equation method [29]. Briefly, we performed 10 imputations and averaged the variable estimates to produce a mean estimate. Finally, we verified that the relative efficiency of the imputation for each variable was greater than 95%. We also constructed multivariate models to test the interaction between participant sex and the exposure variable for each outcome. Then, to test the cumulative effect of both maltreatment and victimization on mental health outcomes, compared to peer victimization alone and maltreatment alone, we conducted the same analyses, but changed the reference categories.

Finally, the attributable fraction (AF) was defined as the proportion of each mental health outcome that was attributable to peer victimization or parental maltreatment or both, calculated as follows: AF = [p*(aOR-1)] / [1+ (p*(aOR-1)], where p was the prevalence of the risk factor in our study sample, and aOR was the adjusted odds ratio related to the association between the risk factor and the outcome in the multivariate model after the imputation for missing data.

All analyses were performed with SAS version 9.4. Two-sided *P* values < 0.05 were considered statistically significant.

## Results

Of the 2476 students that fully completed the baseline and childhood adversity questionnaires, 205 were excluded, due to missing data for the outcomes. Therefore, the final analysis included 2271 students. The mean age of the participants was 21.0 years (SD ± 2.6), and about three quarters were female (*n* = 1782; 78.5%). Nearly one third of the students reported at least one mental health problem (*n* = 654; 28.8%); 22.4% (*n* = 509) reported anxiety, 13.6% (*n* = 308) reported depression symptoms, 18.3% (*n* = 415) reported 12-month suicidal ideations, without a lifetime suicide attempt, and 4.0% (*n* = 91) reported suicidal ideations with a suicide attempt. Mental health problems were more frequent among females than among males, including suicidal thoughts without a suicide attempt (18.5% vs. 17.6%), suicidal thoughts with a suicide attempt (4.2% vs. 3.5%), depression (15.0% vs. 8.4%), and anxiety (25.1% vs. 12.5%). Table 1 shows the characteristics of participants in the overall sample and in the maltreatment and/or peer victimization groups. Nearly one third of the sample (*n* = 677; 29.8%) was exposed to peer victimization alone; 7.5% (*n* = 170) was exposed to maltreatment alone; and 10.3% (*n* = 233) was exposed to both maltreatment and peer victimization. Participants in the two latter groups were more likely to declare negative family events, such as parental divorce, parental depression history, or parental alcohol abuse history, compared to participants in the groups that reported either no adversity or peer victimization alone.

**Table 1:** Characteristics of the study sample overall and according to parental maltreatment or peer victimization

	All sample (***N*** = 2271)		Parental maltreatment or Peer victimization								P-value*
			None (***N*** = 1191; 52.4%)		Peer victimization only (***N*** = 677; 29.8%)		Parental maltreatment only (***N*** = 170; 7.5%)		Both (***N*** = 233; 10.3%)		
**Age (Mean, SD)**	21.0	2.6	21.0	2.4	20.8	2.4	21.3	2.4	21.2	3.8	0.170
**Sex**											
Male	489	21.5	265	22.3	152	22.5	24	14.1	48	20.6	0.095
Female	1782	78.5	926	77.8	525	77.6	146	85.9	185	79.4	
**Perceived parental support** ^**a**^											
Strong to extremely strong	1709	76.1	991	83.8	534	79.6	80	48.8	104	45.4	<.0001
Moderate	389	17.3	155	13.1	117	17.4	44	26.8	73	31.9	
Low or None	148	6.6	36	3.1	20	3.0	40	24.4	52	22.7	
**Parental divorce or separation** ^**b**^	674	30.4	314	26.9	191	29.0	72	44.7	97	42.5	<.0001
**Parental education level** ^**c**^											
University studies	1227	54.9	656	55.9	381	57.3	81	48.5	109	47.8	0.024
Non university studies	1007	45.1	518	44.1	284	42.7	86	51.5	119	52.2	
**Parental depression or anxiety history** ^**d**^	897	44.5	390	36.1	275	46.2	91	64.1	141	69.8	<.0001
**Parental alcohol abuse history** ^**e**^	218	10.2	85	7.5	56	8.6	26	16.7	51	25.3	<.0001
**Difficult economic status during childhood** ^**f**^	151	6.7	52	4.4	46	6.8	15	8.8	38	16.3	<.0001

Missing: a = 25; b = 55; c = 38; d = 253; e = 138; f = 1

All data presented as n (%) unless otherwise noted (SD: Standard deviation)

* *P* Values are based on Kruskal-Wallis test for continuous variable and Chi2 test for categorical variables

Table 2 shows the adjusted logistic regression model results for associations between maltreatment and/or peer-victimization and mental health outcomes, after multiple imputations for missing data on the covariates. Relative to participants that did not experience any maltreatment or peer victimization, those that experienced peer victimization alone had increased odds of anxiety (aOR: 1.90; 95% CI: 1.50–2.40), depression (aOR: 1.95; 95% CI: 1.46–2.60), suicidal ideation without a suicide attempt (aOR: 1.62; 95% CI: 1.26–2.09), and suicidal ideation with a suicide attempt (aOR: 2.70; 95% CI: 1.51–4.85). Similar associations were observed for participants that were exposed to maltreatment alone. Participants that experienced both maltreatment and peer victimization were also more likely to present any mental health problems (aOR: 2.94; 95% CI: 2.17–4.00), depression (aOR: 2.63; 95% CI: 1.79–3.86), or suicidal thoughts with a suicide attempt (aOR of 9.19; 95% CI: 4.48–19.92), compared to participants that did not experience either maltreatment or peer victimization.

**Table 2:** Associations between mental health outcomes and parental maltreatment and/or peer victimization (N = 2271)

Maltreatment or Peer victimization	Suicidal thoughts without attempt		Suicidal thoughts with attempt		Depression		Anxiety		Any mental health problems	
	n	OR (95% CI)	n	OR (95% CI)	n	OR (95% CI)	n	OR (95% CI)	n	OR (95% CI)
None	162	1(reference)	20	1(reference)	109	1(reference)	188	1(reference)	246	1(reference)
Peer victimization only	140	1.62 (1.26–2.09)	29	2.70 (1.51–4.85)	112	1.95 (1.46–2.60)	183	1.90 (1.50–2.40)	225	1.84 (1.48–2.29)
Maltreatment only	45	2.08 (1.41–3.07)	8	2.54 (1.08–6.00)	31	1.85 (1.18–2.91)	54	2.16 (1.49–3.13)	69	2.18 (1.54–3.09)
Both	68	2.64 (1.86–3.76)	34	9.19 (4.98–16.92)	56	2.63 (1.79–3.86)	84	2.62 (1.89–3.64)	114	2.94 (2.17–4.00)

OR: odds ratio, CI: confidence interval

Adjusted for age, sex, parental divorce or separation, difficult economic status during childhood, parental depression or anxiety history, parental alcohol abuse history and parental education level after multiple imputation

Additionally, we found an interaction between exposure to interpersonal violence and the participant’s sex (*p* < 0.0001). However, due to the small number of males in some exposure categories, we could not stratify the analyses by sex.

Table 3 shows the mental health outcomes in the group exposed to both maltreatment and peer victimization compared to the groups exposed to either peer victimization or maltreatment alone. The outcomes were not significantly different between participants that experienced maltreatment combined with peer victimization and those that experienced maltreatment alone, except for suicidal thoughts with suicide attempts (aOR: 3.65; 95% CI: 1.60–8.33). Compared to peer victimization alone, the combination of maltreatment and peer victimization showed significantly stronger associations with any mental health problems (aOR: 1.60; 95% CI: 1.17–2.19) and suicidal thoughts, with or without suicide attempts. In contrast, these two exposure groups showed similar associations with depression and anxiety.

**Table 3:** Associations between mental health outcomes and parental maltreatment only or peer victimization only vs both maltreatment and peer victimization

Maltreatment or Peer victimization vs Both	Suicidal thoughts without attempt		Suicidal thoughts with attempt		Depression		Anxiety		Any mental health problems	
	n	OR (95% CI)	n	OR (95% CI)	n	OR (95% CI)	n	OR (95% CI)	n	OR (95% CI)
Model 1 (*N* = 910)										
Peer victimization only	140	1(reference)	29	1(reference)	112	1(reference)	183	1(reference)	225	1(reference)
Both	68	1.64 (1.18–2.35)	34	3.41 (1.95–5.97)	56	1.35 (0.92–1.98)	84	1.38 (0.99–1.93)	114	1.60 (1.17–2.19)
Model 2 (*N* = 403)										
Maltreatment only	45	1(reference)	8	1(reference)	31	1(reference)	54	1(reference)	69	1(reference)
Both	68	1.28 (0.81–2.03)	34	3.65 (1.60–8.33)	56	1.41 (0.86–2.35)	84	1.22 (0.79–1.87)	114	1.35 (0.90–2.03)

*OR* odds ratio, *CI*: confidence interval

Adjusted for age, sex, parental divorce or separation, difficult economic status during childhood, parental depression or anxiety history, parental alcohol abuse history and parental education level after multiple imputation

The AFs ranged from 6.0 to 45.8% (Table 4) in our study sample. We observed that, for the outcome of any mental health disorders, the AF for peer victimization alone was larger than the AF for maltreatment alone. The AFs for peer victimization alone were closer to the AFs for combined maltreatment and victimization than to the AFs for maltreatment alone. For example, 20.0% of any mental health disorder and 15.6% of suicidal ideation without a suicide attempt could be attributed to peer victimization. In comparison, the corresponding AFs for maltreatment combined with peer victimization were 16.7% and 14.5%, respectively, and the AFs for maltreatment alone were 8.1% and 7.5%, respectively.

**Table 4:** Attributable fractions (%) of parental maltreatment and/or peer victimization for mental health outcomes

	Suicidal thoughts without attempt	Suicidal thoughts with attempt	Depression	Anxiety	Any mental health problems
Peer victimization only	15.6	33.6	22.0	21.1	20.0
Maltreatment only	7.5	10.4	6.0	8.0	8.1
Both	14.5	45.8	14.4	14.3	16.7

## Discussion

### Main findings and interpretation

In this cross-sectional study of 2271 young French adults, we found an increased risk of mental health problems, including anxiety, depression, and STBs, in individuals that had been victimized by peers, whether or not they were exposed to parental maltreatment in childhood. Among individuals that were exposed to both maltreatment and peer victimization during childhood, depression and anxiety did not occur significantly more frequently than those exposed to either maltreatment or peer victimization alone. However, the combined exposure significantly increased the risk of suicidal thoughts with suicide attempts, compared to either exposure alone.

The prevalence of peer victimization and maltreatment in our sample were close to those estimated in previous studies [2, 10–12]. Peer victimization alone was highly prevalent (one third of the sample), which explained the large AFs, comparable to the AFs of the combination of maltreatment and peer victimization.

Our results suggested that peer victimization played an important, independent role in mental health problems, consistent with findings in other studies [30]. A previous longitudinal study highlighted the finding that peer victimization had an independent effect on the mental health of young adults, and the long-term adverse effects on mental health were worse than the effects from maltreatment [31]. Previous studies also showed that peer victimization had an important effect on mental health problems; indeed, the risk of negative mental health outcomes was similar between children that had experienced bullying victimization and children that were placed in institutional or foster care [32].

Studies have shown that parental support and a positive familial relationship in childhood have positive long-term effects on adult mental health and provide a crucial foundation for social interactions [33, 34]. Thus, parental maltreatment in childhood increases the likelihood that a child will be victimized by peers, due to the child’s impaired emotional regulation skills [35]. Indeed, attachment theory highlights the fact children internalize aspects of caregiving relationships, and this experience can later impair peer interactions [36]. It is worth noting that maltreatment and peer victimization refer to experiences of interpersonal violence that occurs in two different environmental contexts – the home and the school, respectively. It is possible that some individuals that experience maltreatment in the home environment see themselves as victims, regardless of the context. However, our results showed that associations between peer victimization and mental health outcomes were not fully explained by exposure to both these types of interpersonal violence. Moreover, these associations remained significant, even after controlling for important familial variables. This finding emphasized the importance of interpersonal experiences that occur outside the family sphere, and specifically with peers, which appear to play a specific role in mental health. As children grow-up, peer interactions become increasingly important. Repetitive verbal or physical harassment or exclusion from peers might have long-term effects, independent of other childhood adversities, and thus, they can modify responses to stress [37]. Furthermore, our findings suggested that cumulative maltreatment effects may be at least partly due to victimization. Thus, future studies of maltreatment should also take into account peer victimization.

In suicide ideation-to-action frameworks, such as the Interpersonal Psychological Theory of Suicide [38], or more recently, the Three-Step Theory [39], the development of suicide ideation and its progression to suicide attempts are distinct processes with different explanations and risk factors. These theories emphasize an ‘acquired capability’, which refers to an individual’s habituation to pain or fear through exposure to life experiences, such as abuse or other painful events. Our findings showed that cumulative exposures to parental maltreatment and peer victimization were more strongly associated with the risk of suicidal ideation and suicide attempts than with the risk of suicide ideation alone. This finding suggested that cumulative experiences could promote progression of the acquired capability from suicide ideation to suicide attempts.

### Strengths and limitations

The present study had some important strengths, including the large, contemporaneous sample of young French adults, a study population never investigated on this subject. We also note the consistency of our results with previous studies, and the ability to adjust for a range of confounders, due to the data available on a wide range of variables. Depression and anxiety were measured with validated scales that had excellent psychometric properties. Furthermore, we found a significant association between peer victimization alone and each mental health outcome, which provided consistent results. Despite these strengths, several limitations should be considered when interpreting the results. First, the study design was cross-sectional; therefore, we could not strictly correlate the exposure timing to the outcome and the covariates, and we could not infer causality between the exposure and the mental health outcomes. Second, the voluntary participation of students in the baseline and supplementary questionnaires may have introduced a self-selection bias, although it is difficult to predict how this potential bias could have influenced the results. Third, the information was self-reported, which could lead to an information bias, particularly a memory bias, because the exposure assessment was retrospective. Fourth, there was an over-representation of women in our sample, compared to the 56% of female students in France, and our results suggested that there were differences between the sexes, in terms of mental health problems and maltreatment exposure. Fifth, we did not analyze physical and psychological maltreatments separately, with regard to peer victimization. Finally, we could not investigate the age of onset for peer victimization and/or maltreatment.

## Conclusions

This study showed that, in a large sample of young adults, exposures to parental maltreatment and/or peer victimization in childhood or adolescence were associated with an increased risk of mental health problems. Peer victimization appeared to play a specific role in mental health problems, beyond the role of exposure to maltreatment. Peer victimization occurs frequently, but it is preventable. Therefore, our findings stress the importance of coordinating the efforts of schools, teachers, health services, and public policies in addressing this widespread problem.

## Data Availability

The datasets used and/or analyzed during the current study are available from the corresponding author on reasonable request.

## Abbreviations

AF: Attributable fraction
aOR: Adjusted odds ratio
CI: Confidence interval
i-Share: Internet-based Students’ Health Research Enterprise
OR: Odds ratio
PHQ-9: Patient Health Questionnaire
SD: Standard deviation
STAI: State-Trait Anxiety Inventory
STB: Suicidal thoughts and behaviors
